# Effects and potential mechanisms of *si-ni-san* on animal models of depression: a preclinical systematic review and meta-analysis

**DOI:** 10.3389/fphar.2026.1818197

**Published:** 2026-10-08

**Authors:** Yuzhi Wang, Qun Wang, Hui Xie, Xiaozhu Liu, Yuhuan Ju, Xifeng Liu, Shiguang Sun

**Affiliations:** 1 College of Pharmacy, Shandong University of Traditional Chinese Medicine, Jinan, China; 2 College of Traditional Chinese Medicine, Shandong University of Traditional Chinese Medicine, Jinan, China; 3 Department of Vertigo, Jinan Shizhong People’s Hospital, Jinan, China; 4 Department of Pharmacy, Second Affiliated Hospital, Shandong Provincial Hospital of Integrative Medicine, Shandong University of Traditional Chinese Medicine, Jinan, China

**Keywords:** depression, meta-analysis, potential mechanism, preclinical studies, *Si-ni-san*, systematic review

## Abstract

**Context:**

In Traditional Chinese Medicine depressive disorders are commonly associated with liver qi stagnation syndrome. *Si-ni-san* (SNS), a classic formula for soothing the liver and relieving qi stagnation, is widely used for its potential antidepressant-like effects.

**Objective:**

This preclinical systematic review and meta-analysis aimed to evaluate the pharmacological effects of SNS on depression-related behavioral outcomes in animal models and to summarize its mechanisms of action.

**Methods:**

We conducted a systematic search of seven databases (including PubMed) for records published up to December 2025. Risk of bias was assessed using SYRCLE’s tool. Meta-analyses were performed using STATA 15.0 and RevMan 5.4. Subgroup and leave-one-out sensitivity analyses were conducted to test the robustness of the findings. Publication bias was assessed using funnel plots and Egger’s test. Trim-and-fill analysis was performed to adjust for potential publication bias. The certainty of evidence was evaluated using a modified GRADE approach for preclinical studies.

**Results:**

A total of 28 studies were included, involving 384 animals and three behavioral tests: sucrose preference test (SPT), forced swim test (FST), and tail suspension test (TST). SNS treatment was associated with increased sucrose preference in the SPT (SMD = 3.18, 95% CI = [2.44, 3.93]), and reduced immobility time in both the FST (SMD = −2.17, 95% CI = [-2.86, −1.49]) and TST (SMD = −2.55, 95% CI = [-3.31, −1.78]). Mechanistically, SNS treatment was associated with increased monoamine neurotransmitters (MNTs), reduced hypothalamic-pituitary-adrenal (HPA) axis hormones, and decreased pro-inflammatory cytokines.

**Conclusion:**

These findings suggest that SNS is associated with antidepressant-like pharmacological effects in rodent models, with changes in MNTs, HPA axis hormones, and inflammatory markers. However, due to substantial heterogeneity, risk of bias, and inadequate phytochemical characterization, these results should be considered hypothesis-generating and interpreted with caution.

## 1 Introduction

Depression is a prevalent mental disorder characterized by persistent low mood and anhedonia, which significantly reduces quality of life and imposes a substantial global disease burden (Malhi et al., 2026). Data from the Global Burden of Disease Study 2023, indicate the age-standardized prevalence rate of major depressive disorder was 2,815.8 per 100,000 population, making it a leading contributor to worldwide disability (Mental Disorder Collaborators, 2026). Depression has multifactorial risk factors, including dysfunctional cognitions, stressful life events, adverse circumstances, a family history of depression, interpersonal dysfunction, and female sex (Hammen, 2018). Furthermore, the frequent comorbidity of depression with other chronic conditions often exacerbates their health outcomes, highlighting the critical need to prioritize its management in public health to mitigate the overall burden of disease and disability (Moussavi et al., 2007).

Given the substantial burden of depression, elucidating its underlying pathogenesis is essential for developing more effective and targeted therapeutic strategies (Cui et al., 2024). The pathogenesis of depression is multifactorial, with growing evidence implicating monoaminergic dysfunction, hypothalamic-pituitary-adrenal (HPA) axis hyperactivity, and neuroinflammation as core pathophysiological mechanisms (Kajumba et al., 2024). These pathophysiological mechanisms directly inform therapeutic development. The monoamine hypothesis, for example, prompted the development of selective serotonin reuptake inhibitors (SSRIs), which continue to serve as first-line pharmacotherapy for depression (Singh et al., 2025). Nevertheless, SSRIs exclusively target monoamine reuptake, while largely neglecting other critical pathways, including neuroinflammation and HPA axis dysregulation (Bose et al., 2026). Substantial evidence also indicates a range of adverse effects associated with these antidepressants, including gastrointestinal disturbances, dry mouth, hepatotoxicity, sexual dysfunction, seizures, abnormal bleeding, and weight changes (Wang et al., 2018). These limitations underscore the need for alternative interventions, such as traditional medicine formulas that can simultaneously target multiple pathogenic pathways.

Traditional Chinese Medicine (TCM) plays an increasingly prominent role in the management of depression (Zhuang et al., 2023). *Si-ni-san* (SNS) comprises *Bupleurum chinense* DC [Apiaceae; Bupleuri Radix], *Paeonia lactiflora* Pall [Ranunculaceae; Paeoniae Radix Alba], *Citrus aurantium* L [Rutaceae; Aurantii Fructus Immaturus], and *Glycyrrhiza uralensis* Fisch [Fabaceae; Glycyrrhizae Radix et Rhizoma] and is used to treat depression. This formulation was first described in the ancient text *Shanghan Lun* (Treatise on Cold Damage Disorders), written by the Eastern Han Dynasty physician *Zhang Zhongjing* (150–219 A.D.). According to TCM theory, the liver governs the smooth flow of qi and regulates emotional activities, and liver qi stagnation is considered a core pathological basis of depression. SNS, a classic formula for soothing the liver and relieving qi stagnation, has been used to treat conditions characterized by emotional distress. From a modern biomedical perspective, this state often correlates with dysregulation of monoamine neurotransmitters (MNTs), hyperactivation of the HPA axis, and chronic neuroinflammation (Li X et al., 2020; Zhang et al., 2024). Converging preclinical evidence suggests the antidepressant-like potential of SNS. A systematic review comprehensively summarized preclinical experimental studies demonstrating that SNS regulates the HPA axis, suppresses neuroinflammation, and restores monoamine neurotransmitter levels in rodent models (Shen et al., 2023). In addition, a review has suggested that SNS exerts antidepressant-like effects, potentially mediated by mechanisms involving neurological modulation, neurotransmitter regulation, inflammatory response regulation, and neuroendocrine adjustments (Wang et al., 2015). However, no comprehensive preclinical meta-analysis on SNS for depression has been conducted. Therefore, we conducted this meta-analysis to evaluate the overall pharmacological effects of SNS on depression-related behavioral outcomes in rodent models and to summarize its potential mechanisms of action.

## 2 Materials and methods

We followed the guidelines of the Preferred Reporting Items for Systematic reviews and Meta-Analyses (PRISMA) statement (Page et al., 2021). This study was registered with PROSPERO (CRD42022361895).

### 2.1 Search strategy

Two reviewers independently conducted a systematic literature search on the treatment of depression with SNS. The systematic search was performed in PubMed, Embase, Web of Science, China National Knowledge Infrastructure (CNKI), Wan Fang Database, Chinese Scientific Journal Database (VIP), and Chinese Biological Medicine Database (SinoMed) from inception to December 2025, with no language restrictions. The search strategy employed the terms: “*Sini*”, “*Sinisan*”, “*Sini San*”, “*Sini* Powder”, “depression”, “animal model”, and “experimental animal model”, as well as their respective abbreviations, and all synonymous variants for each database. The complete search strategy is available in the Supplementary Material.

### 2.2 Inclusion criteria and exclusion criteria

The eligibility criteria were defined according to the PICOS framework. 1) Participants (P): animal models of depression, including chronic mild stress (CMS), chronic restraint stress (CRS), maternal separation (MS), or lipopolysaccharide (LPS)-induced models. In addition, studies using acute behavioral despair (BD) tests specifically the forced swimming test (FST) and tail suspension test (TST) as screening paradigms were also included. These tests are not disease models *per se* but are widely used as behavioral assays to evaluate antidepressant-like effects. 2) Interventions (I): SNS treatment, regardless of dose ratio, dosage, and duration; 3) Comparators (C): no treatment or vehicle treatment; 4) Outcomes (O): (a) behavioral outcomes were assessed using three validated paradigms: the sucrose preference test (SPT; outcome: sucrose preference percentage and consumption), FST (outcome: immobility time), and TST (outcome: immobility time); (b) mechanistic outcomes: any molecular measure related to monoamine neurotransmitters, HPA axis, inflammatory factors, synaptic plasticity, or oxidative stress; 5) Study designs (S): experimental animal studies.

The exclusion criteria were as follows: 1) non-original studies (e.g., reviews, conference abstracts), studies without a separate model group; 2) non-animal studies (*in vitro*, *ex vivo*, or human); 3) studies lacking behavioral outcome data (SPT, FST, or TST).

### 2.3 Literature screening and data extraction

References were managed using EndNote 20. Duplicates were removed using EndNote’s automatic duplicate detection function followed by manual checking. Two independent investigators (YZ Wang and Q Wang) screened the studies. First, titles and abstracts were screened for preliminary eligibility. Subsequently, the full texts of potentially relevant studies were reviewed to confirm final inclusion. The screening process was documented in a PRISMA flow diagram. For non-journal sources, dissertations were included if they met the criteria and conference abstracts were excluded due to insufficient data. For duplicate publications from the same experiment, we retained the most complete dataset. Data extraction from the included studies covered the following: study characteristics (authors, publication year); animal characteristics (species, strain, sex, weight, and sample size); model characteristics (depression model type and model duration); intervention characteristics (SNS formulation, dosage, administration route, administration time, quality control and source); and outcome measures (behavioral tests including SPT, FST, and TST, along with relevant molecular mechanisms). In addition, safety-related data were systematically extracted from the included studies, including mortality, adverse behavioral effects, organ toxicity, and any mention of drug interactions. Any disagreements were resolved by the third author (H Xie).

### 2.4 Characterization of intervention reporting quality

To evaluate the completeness of reporting for the SNS preparation, we adapted criteria from the Consensus statement on the Phytochemical Characterization of Medicinal Plant extracts (ConPhyMP) guidelines (Heinrich et al., 2022) and classified each included study into one of three categories. 1) Adequately characterized: The study reported the preparation form, detailed extraction method, and at least two of the following: botanical authentication (with Latin name or voucher specimen), batch information, pharmacopeial compliance, or chemical characterization (high-performance liquid chromatography (HPLC) fingerprinting or marker quantification). These criteria reflect the minimum information needed to reasonably replicate the intervention; 2) Partially characterized: The study reported the preparation form and extraction method, but lacked authentication, batch information, and chemical characterization. The basic preparation method is known, but the specific material used is not fully traceable; 3) Inadequately characterized: The study reported only the formula name with no or minimal details on preparation, sourcing, or quality control. The intervention cannot be meaningfully replicated based on the information provided. For each included study, we extracted information on botanical name, plant part, formula ratio, source or manufacturer, batch number, authentication or voucher specimen, preparation or extraction method, pharmacopeial compliance, chemical fingerprinting, marker quantification, and analytical methods.

We also assessed the included studies against the “Four Pillars of Best Practice” framework as defined by Frontiers in Pharmacology for botanical research. Pillar 1 (pharmacological requirements) assessed whether the study: 1) described the traditional context with primary references; 2) used a reasonable dose range with appropriate controls and reported basic pharmacological data (i.e., tested at least two dose levels and included a positive control); and 3) employed credible experimental models (e.g., CMS, CRS). Studies meeting all three criteria were considered compliant. Pillar 2 (composition requirements) assessed whether the study adequately characterized the SNS preparation based on the three-tier classification described above: studies classified as adequately characterized were considered fully compliant; partially characterized as partially compliant; and inadequately characterized as non-compliant. Pillar 3 (ethical requirements) assessed whether the study explicitly reported ethical approval from an institutional animal care and use committee or equivalent review body, including the approval number where available. Studies reporting such approval were considered compliant.

### 2.5 Risk of bias

The risk of bias of the included studies was assessed independently by two reviewers (YZ Wang and Q Wang) using the SYRCLE’s risk of bias tool (Hooijmans et al., 2014). The tool assesses the following domains: Selection bias (random sequence generation, baseline characteristics, allocation concealment); Performance bias (random housing, blinding of participants and personnel); Detection bias (random outcome assessment and blinding of outcome assessment); Attrition bias (incomplete outcome data); Reporting bias (selective reporting); Other bias. Following SYRCLE’s guidance, each domain was judged as “low risk”, “high risk”, or “unclear risk” based on the reported methodological information. When insufficient details were reported to permit a definitive judgment, “unclear risk” was assigned, as the absence of reporting does not necessarily indicate inadequate methodology. “High risk” was assigned only when the methods were explicitly reported as inadequate or flawed. Any disagreement was resolved by a third reviewer (H Xie).

### 2.6 Statistical analysis

A random-effects meta-analysis was conducted in STATA 15.0, with continuous outcomes expressed as standardized mean differences (SMD; Hedges’ g) and 95% confidence intervals (CI). Data extraction from figures was performed using WebPlotDigitizer 4.5. Heterogeneity was quantified using the *I* ^*2*^ statistic, and subgroup analyses were conducted to explore potential sources. Sensitivity analyses were performed to test the robustness of the findings. Specifically, we conducted leave-one-out sensitivity analyses by sequentially excluding each individual study and recalculating the pooled effect size for each of the three primary outcomes: SPT, FST, and TST. A study was considered influential if its exclusion resulted in a substantial change in the pooled SMD or 95% CI. Publication bias was assessed for outcomes with 10 or more included studies using funnel plots and Egger’s test. Funnel plot asymmetry was visually inspected, and Egger’s test was used to quantitatively assess asymmetry (*P* < 0.05 indicating significant publication bias). For outcomes with fewer than 10 studies, publication bias was not formally assessed due to the low statistical power of funnel plot-based methods in small meta-analyses. To assess the potential impact of publication bias on the robustness of our findings, we performed trim-and-fill analysis. The results of the risk of bias assessment were recorded and visualized using RevMan 5.4.

### 2.7 GRADE certainty of the evidence

The certainty of evidence was assessed using the Grading of Recommendations Assessment, Development and Evaluation (GRADE) approach for preclinical animal studies (Hooijmans et al., 2018). Two reviewers independently rated the certainty for each outcome as high, moderate, low, or very low based on five domains: risk of bias (SYRCLE tool), indirectness (animal-to-human translation), inconsistency (statistical heterogeneity), imprecision (confidence intervals and sample size), and publication bias (funnel plot or Egger’s test). The online software GRADEpro was used to record and organize the certainty assessments.

## 3 Results

### 3.1 Search results

After database retrieval, 608 citations were identified in seven databases. After reviewing the full texts, 28 studies (Chang, 2003; Jin, 2004; Wang et al., 2004; Yu and Jin, 2009; Li et al., 2013; Yi et al., 2013; Li and Xu, 2014; Peng, 2014; Zhou et al., 2015; Hu et al., 2016; Wei et al., 2016; Wang, 2017; Cao, 2019; Ni, 2019; Su, 2019; Shen et al., 2020; Cui, 2021; Li et al., 2021; Deng et al., 2022; Wang W et al., 2022; Yao et al., 2022; Fang et al., 2023; Yang, 2023; Zhang et al., 2023; Liu, 2024; Cai et al., 2025; Du et al., 2025; Wang et al., 2025) published between 2003 and 2025 were ultimately included. The selection process is detailed in Figure 1.

**FIGURE 1:**
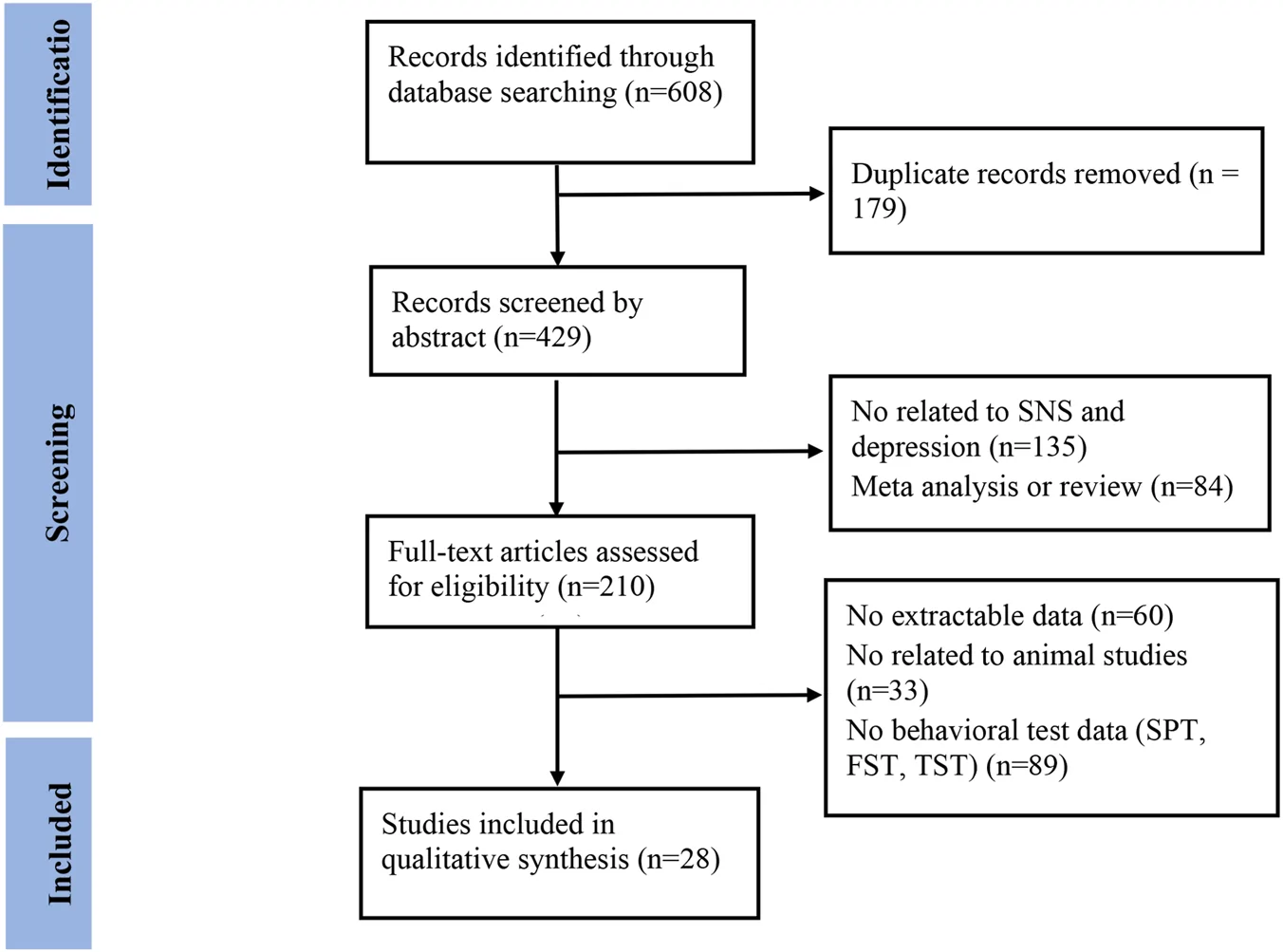
The flowchart of papers inclusion process.

### 3.2 Study characteristics

Regarding the animal characteristics, 72% (n = 20) used rats and 28% (n = 8) used mice (Figure 2A). One study used both male and female mice, one did not report the sex of the animals, and all others used male animals only. Four depression models were used across the studies: CMS (61%, n = 17), MS (14%, n = 4), LPS (7%, n = 2), and MS + CMS (3%, n = 1), MS + CRS (3%, n = 1). In addition, three studies (12%) used BD paradigms (Figure 2B). In terms of interventions, the interventions were administered with treatment durations of 1–14 days (22%, n = 6), 14–28 days (43%, n = 12), 28–42 days (28%, n = 8), 42–56 days (7%, n = 2) (Figure 2C). The primary outcomes derived from behavioral tests were sucrose preference percentage and consumption (SPT) and immobility time (FST and TST). The tests were distributed as follows: SPT (39%, n = 11), FST alone (7%, n = 2), FST + TST (18%, n = 5), SPT + FST (25%, n = 7), and all three tests (11%, n = 3) (Figure 2D). The detailed results of the study characteristics are shown in Table 1.

**FIGURE 2:**
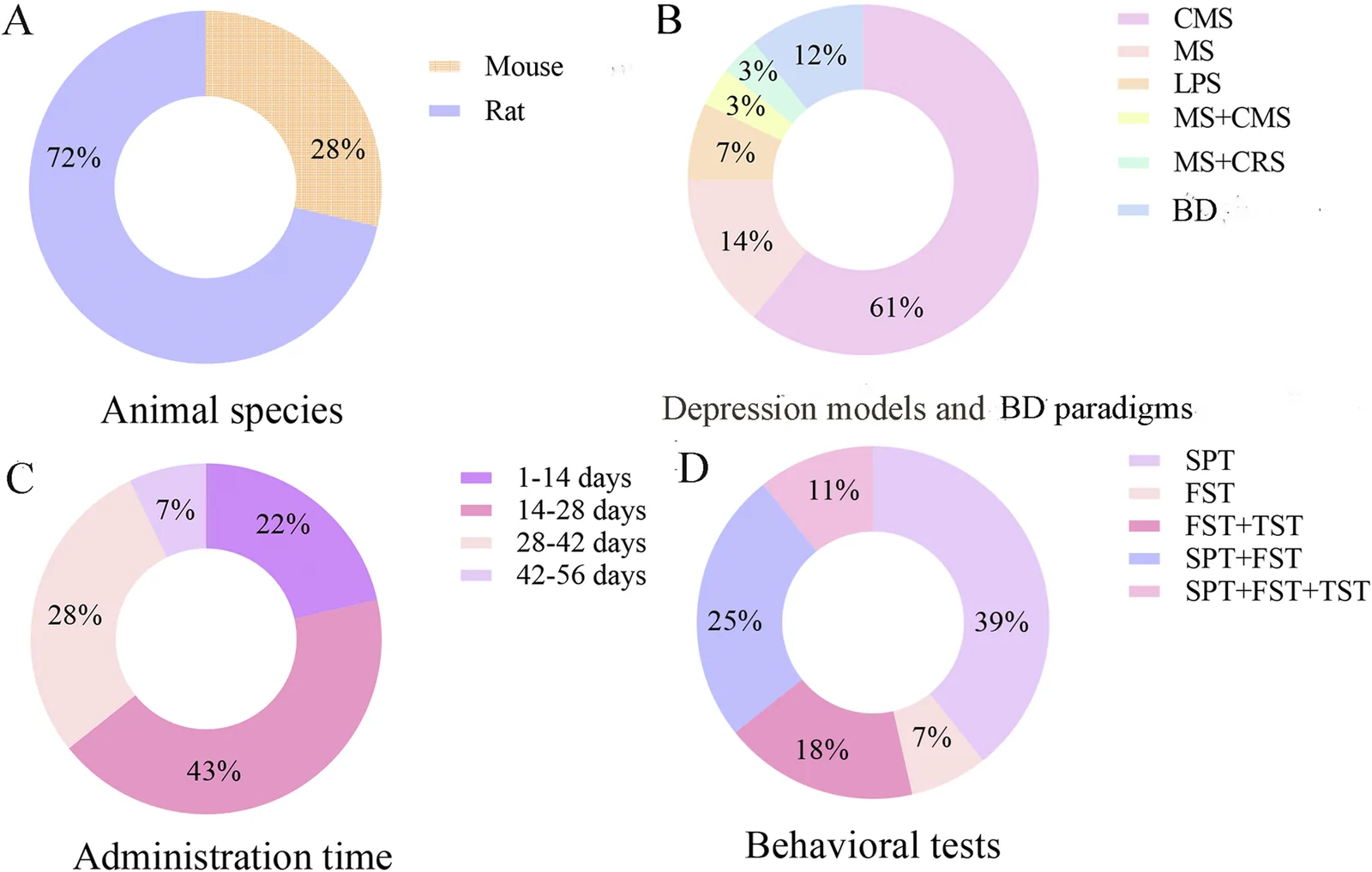
Included study characteristics **(A)** Animal species; **(B)** Depression models and BD paradigms **(C)** Administration time; **(D)** Behavioral tests.

**TABLE 1:** The main characteristics of the included studies.

Study	Animal characteristics					Model characteristics		Intervention characteristics				Outcome measures			
	Species	Strain	Sex	Weight	N (C/I)	Animal model or screening paradigm	Duration	Form	Dosage	Administration time	Quality control and source	SPT	FST	TST	Safety assessment reported
Zhou et al., 2015	Mouse	ICR	Male	32–38 g	8/8	LPS	1 day	Granules	0.8 g/kg	10 days	NR, R	​	√	​	NR
Su 2019	Mouse	ICR	Male	20.6–23 g	10/10	LPS	1 day	Water decoction	0.04 g/kg	7 days	NR, NR	​	√	√	NR
Wang et al., 2004	Mouse	KM	Male	20–25 g	15/15	BD	NR	Water decoction	20 g/kg	14 days	NR, R	​	√	√	NR
Yi et al., 2013	Mouse	KM	Male	20–24 g	10/10	BD	NR	Water extraction	1.3 g/kg	1 day	NR, R	​	√	√	NR
Zhang et al., 2023	Mouse	C57BL/6	Male	18–22 g	12/12	CMS	42 days	Decoction	19.6 g/kg	21 days	R, R	√	√	√	NR
Cai et al., 2025	Mouse	C57BL/6	Male	16–20 g	6/6	CMS	42 days	Water decoction	20.8 g/kg	21 days	NR, R	​	√	√	NR
Du et al., 2025	Mouse	C57BL/6	Male	18–22 g	15/15	CMS	28 days	Water decoction	19.6 g/kg	28 days	NR, R	√	√	√	NR
Liu 2024	Mouse	C57BL/6	Male	NR	10/10	MS + CMS	28 days	Water decoction	10 g/kg	28 days	NR, R	√	√	√	NR
Peng 2014	Rat	Wistar	Male	160–200 g	10/10	CMS	28 days	Water decoction	6.8 g/kg	56 days	NR, R	√	​	​	NR
Hu et al., 2016	Rat	Wistar	Male	180–220 g	10/10	CMS	30 days	Granules	29.67 g/kg	30 days	NR, R	√	​	​	NR
Wang 2017	Rat	Wistar	Male	130–150 g	12/12	CMS	32 days	Granules	0.0027 g/kg	35 days	NR, R	√	√	​	NR
Ni 2019	Rat	Wistar	Male	180–220 g	15/15	CMS	42 days	Granules	5 g/kg	42 days	NR, R	√	​	​	NR
Shen et al., 2020	Rat	Wistar	Both	NR	12/12	MS + CMS	21 days	Water extraction	10 g/kg	40 days	R, R	√	√	​	NR
Chang 2003	Rat	SD	Male	180–200 g	17/17	CMS	28 days	Water decoction	20 g/kg	21 days	NR, R	​	√	√	NR
Jin 2004	Rat	SD	Male	180–200 g	6/6	CMS	28 days	Water decoction	20 g/kg	27 days	NR, R	√	​	​	NR
Yu and Jin, 2009	Rat	SD	Male	180–220 g	8/8	CMS	21 days	Granules	10 g/kg	21 days	NR, R	√	√	​	NR
Li et al., 2013	Rat	SD	Male	190–210 g	40/40	CMS	28 days	Ethanol extraction	0.48 g/kg	28 days	NR, R	√	​	​	NR
Li et al., 2014	Rat	SD	Male	100–200 g	10/10	CMS	21 days	Water decoction	10 g/kg	42 days	NR, R	√	​	​	NR
Li et al., 2021	Rat	SD	Male	180–220 g	20/20	CMS	21 days	Granules	5 g/kg	21 days	NR, R	√	​	​	NR
Yao et al., 2022	Rat	SD	Male	200–250 g	10/10	CMS	28 days	Water decoction	0.0128 g/kg	28 days	NR, R	√	​	​	NR
Wang W et al., 2022	Rat	SD	Male	180–220 g	10/10	CMS	42 days	Granules	5 g/kg	21 days	NR, R	√	​	​	NR
Fang et al., 2023	Rat	SD	Male	250–270 g	20/20	CMS	28 days	Water decoction	5.32 g/ml	14 days	NR, R	√	​	​	NR
Yang 2023	Rat	SD	Male	180–220 g	12/12	CMS	41 days	Water decoction	5 g/kg	21 days	NR, R	√	​	​	NR
Deng et al., 2022	Rat	SD	Male	NR	8/8	MS	21 days	Water decoction	10 g/kg	30 days	R, R	√	√	​	NR
Cui 2021	Rat	SD	Male	NR	12/12	MS	21 days	Water decoction	10 g/kg	30 days	NR, R	√	√	​	NR
Cao 2019	Rat	SD	Male	NR	6/6	MS	21 days	Water decoction	10 g/kg	53 days	R, R	√	√	​	NR
Wang et al., 2025	Rat	SD	Male	NR	12/12	MS	21 days	Water decoction	10 g/kg	17 days	NR, NR	√	√	​	NR
Wei et al., 2016	Rat	SD	Male	180–220 g	7/7	BD	NR	Ethanol extraction	4 g/kg	7 days	R, R	​	√	​	NR

C, control group; I, intervention group; NR, no reported, R reported, SPT, the sucrose preference test; FST, the forced swimming test; TST, the tail suspension test; CMS, chronic mild stress; CRS, chronic restraint stress; MS, maternal separation; LPS, lipopolysaccharide; BD, behavioral despair.

### 3.3 Risk of bias

We conducted a quality assessment of the risk of bias methodology in 28 studies. Among the 28 included studies, eight (Yu and Jin, 2009; Li et al., 2013; Yi et al., 2013; Cao, 2019; Wang W et al., 2022; Yao et al., 2022; Yang, 2023; Cai et al., 2025) described their randomization method in detail, while this was not specified in the remaining studies. For baseline characteristics, six studies (Jin, 2004; Yu and Jin, 2009; Peng, 2014; Hu et al., 2016; Yang, 2023; Cai et al., 2025) explicitly reported baseline comparability and were judged as low risk. Similarly, missing data were reported in one study (Ni, 2019); were unclear in three studies (Wang, 2017; Wang W et al., 2022; Du et al., 2025); and were explicitly reported as complete in the remaining studies. For all studies, allocation concealment, blinding of participants and personnel, random outcome assessment, blinding of outcome assessment, and other biases were assessed as unclear. Randomized housing and selective reporting were assessed as low risk. For the “random housing” domain, studies were considered low risk when animals were housed under identical conditions and outcomes were objectively measured. For the “selective outcome reporting” domain, studies were judged as low risk when the results section reported all outcomes prespecified in the methods section. The detailed results of the risk of bias assessment are shown in Figure 3.

**FIGURE 3:**
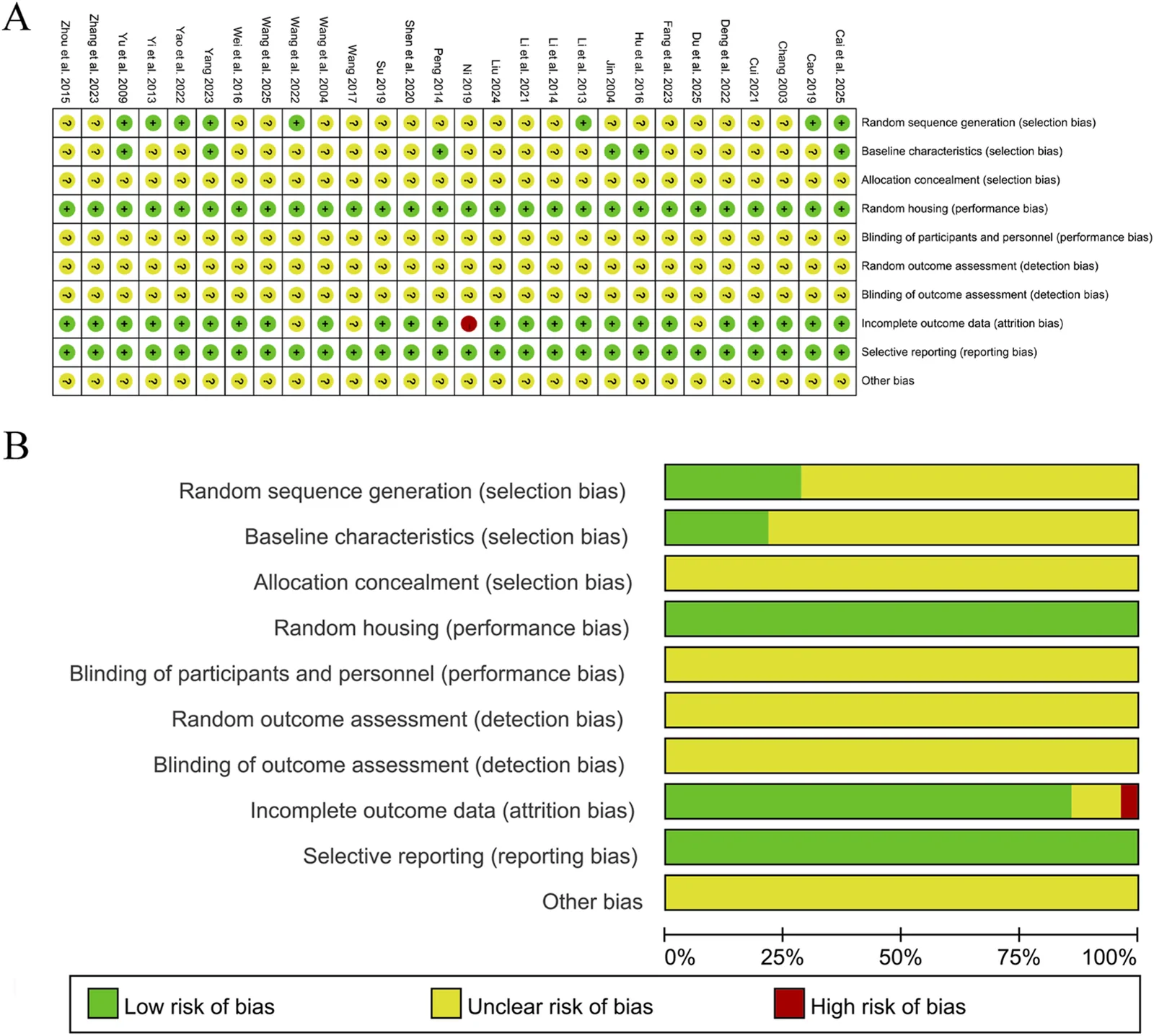
Risk of bias assessment chart **(A)** Risk of bias summary; **(B)** Risk of bias graph.

### 3.4 Meta-analysis

#### 3.4.1 Sucrose preference test

Twenty-one studies involving a total of 275 animals were included in the meta-analysis to assess SPT. The meta-analysis revealed that preference for sucrose was significantly increased in the SNS group compared with the control group (SMD = 3.18, 95% CI = [2.44, 3.93]), with substantial heterogeneity (*I* ^*2*^ = 87.4%, *P* < 0.001) (Figure 4A).

**FIGURE 4:**
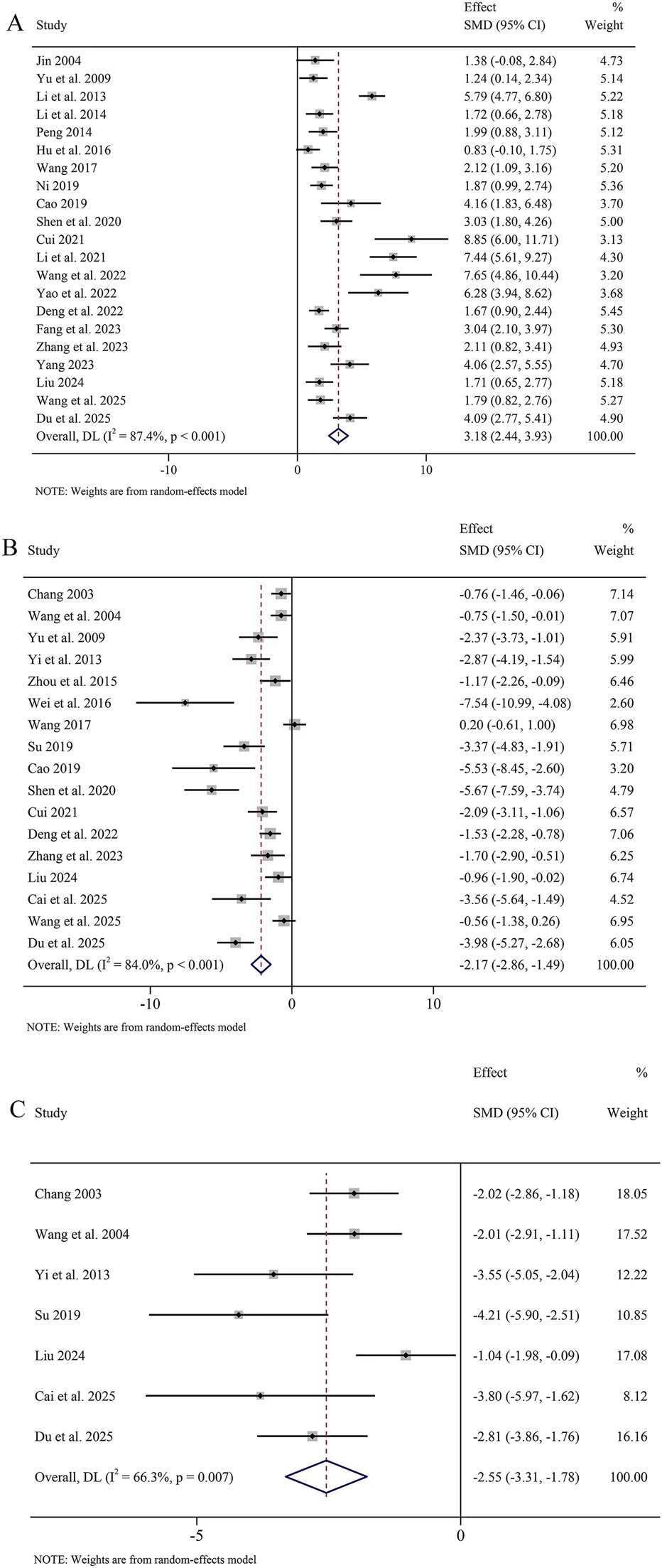
Forest plot for the effect of *Si-ni-san* on **(A)** the sucrose preference test **(B)** the forced swimming test; **(C)** the tail suspension test.

#### 3.4.2 Forced swimming test

Seventeen studies involving a total of 186 animals were included in the meta-analysis to assess FST. The meta-analysis revealed that immobility time was significantly reduced in the SNS group compared with the control group (SMD = −2.17, 95% CI = [-2.86, −1.49]), with substantial heterogeneity (*I* ^*2*^ = 84.0%, *P* < 0.001) (Figure 4B).

#### 3.4.3 Tail suspension test

Seven studies involving a total of 83 animals were included in the meta-analysis to assess TST. The meta-analysis revealed that immobility time was significantly reduced in the SNS group compared with the control group (SMD = −2.55, 95% CI = [-3.31, −1.78]), with moderate heterogeneity (*I* ^*2*^ = 66.3%, *P* = 0.007) (Figure 4C).

#### 3.4.4 Sensitivity analysis, subgroup meta-analysis and publication bias

Leave-one-out sensitivity analyses showed that no single study exerted a significant influence on the pooled effect sizes, as all SMD remained within the 95% CI of the estimates (Supplementary Figure S1).

Substantial statistical heterogeneity was observed across all three behavioral tests: SPT *(I* ^*2*^ = 87.4%, *P* < 0.001), FST (*I* ^*2*^ = 84.0%, *P* < 0.001), and TST (*I* ^*2*^ = 66.3%, *P* = 0.007). To explore potential sources of heterogeneity, we performed subgroup analyses based on animal strain, animal species, depression model or BD, dose, and treatment duration. For the SPT, animal strain was a significant source of heterogeneity (*P* = 0.011), with effect sizes varying notably across strains: SD rats (SMD = 3.98, *I* ^*2*^ = 90.5%), Wistar rats (SMD = 1.90, *I* ^*2*^ = 53.4%), and C57BL/6 mice (SMD = 2.60, *I* ^*2*^ = 75.2%). For the FST (*P* = 0.002) and TST (*P* = 0.007), depression model or BD was a significant contributor. The largest FST effect sizes were observed in the MS + CMS combined model (SMD = −5.67) and the BD (SMD = −3.17), while effects for the CMS (SMD = −1.90), MS (SMD = −1.82), and LPS (SMD = −2.22) models were smaller. For the TST, the LPS model showed the largest effect (SMD = −4.21), followed by the BD (SMD = −2.66) and the CMS model (SMD = −2.55). TST subgroup analysis identified dosage as a significant heterogeneity source (*P* = 0.011), with lower doses (<10 g/kg) showing larger effects (SMD = −3.84) than higher doses (≥10 g/kg) (SMD = −2.11), though this finding was based on only two studies. These results suggest that the animal model or BD, dose, and strain may contribute to heterogeneity (Supplementary Table S1).

Publication bias was assessed for the SPT and FST (which included >10 studies) using funnel plots and Egger’s test. The results indicated the presence of significant publication bias (Egger’s, *P* < 0.05) (Figure 5). For the TST outcome, publication bias was not formally assessed due to the limited power of funnel plot and Egger’s test in meta-analyses with fewer than 10 studies. To further evaluate the impact of publication bias on the reliability of the meta-analysis findings, we performed trim-and-fill analysis for the SPT and FST. For the SPT, trim-and-fill adjustment imputed four potentially missing studies, reducing the effect size from 3.18 to 2.43. Although the adjusted estimate remained statistically significant (adjusted SMD = 2.43, 95% CI = [1.61, 3.25]), this reduction suggests that publication bias may have led to a notable overestimation of the SPT effect. For the FST, no missing studies were imputed, and the adjusted effect remained unchanged (SMD = −2.17, 95% CI = [-2.86, −1.49]), suggesting that publication bias was less of a concern for this outcome (Supplementary Figure S2).

**FIGURE 5:**
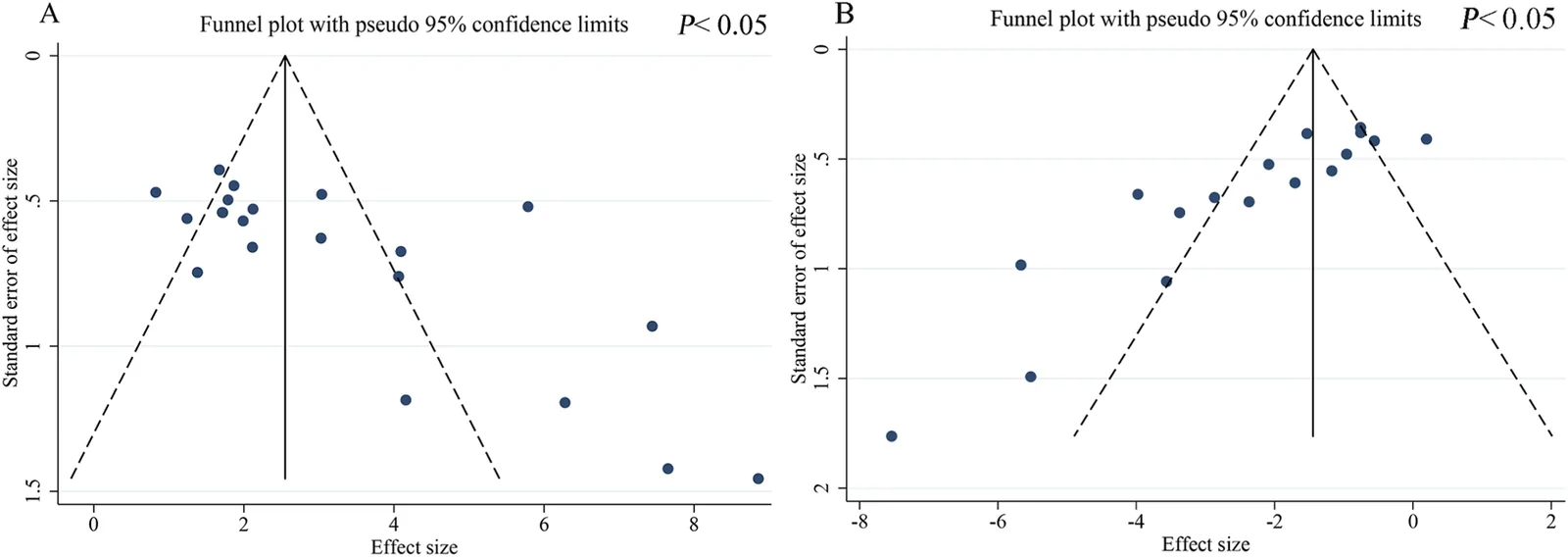
Funnel plots of the included studies **(A)** The sucrose preference test; **(B)** The forced swimming test.

#### 3.4.5 GRADE certainty of the evidence

In terms of risk of bias, most studies had unclear risk due to incomplete reporting, leading to downgrading. In terms of inconsistency, substantial unexplained heterogeneity remained despite consistent direction of effect. Therefore, we downgraded for inconsistency. In terms of indirectness, inherent animal-to-human extrapolation required downgrading by one level. For imprecision, although the confidence intervals did not cross the null line, the total sample sizes were relatively small for outcomes, and the substantial heterogeneity further reduced the precision of the estimates. Therefore, we downgraded one level for imprecision. In terms of publication bias, most studies had publication bias. Based on the GRADE criteria, the certainty of evidence was rated as very low for the SPT, FST and TST. The detailed GRADE certainty assessment is presented in Table 2.

**TABLE 2:** The GRADE certainty level.

Certainty assessment						Certainty
Outcome	Risk of bias	Inconsistency	Indirectness	Imprecision	Other considerations	
SPT	Serious^a^	Serious^b^	Serious^c^	Serious^d^	Publication bias strongly suspected	⊕⃝⃝⃝ Very low
FST	Serious^a^	Serious^b^	Serious^c^	Serious^d^	Publication bias strongly suspected	⊕⃝⃝⃝ Very low
TST	Serious^a^	Serious^b^	Serious^c^	Serious^d^	Publication bias strongly suspected	⊕◯◯◯ Very low

^a^ Most studies presented uncertain risk of bias.

^b^ Statistical heterogeneity (*I^2^* > 50%).

^c^ Extrapolation from animals to humans is inherently indirect.

^d^ the total sample sizes were relatively small; SPT, the sucrose preference test; FST, the forced swimming test; TST, the tail suspension test.

#### 3.4.6 Molecular and biochemical correlates of SNS in depression

The reported molecular and biochemical correlates of SNS treatment in depression are summarized in Table 3 and illustrated in Figure 6. The primary correlates identified across studies include changes in the regulation of MNTs, anti-inflammatory effects, the HPA axis, synaptic plasticity proteins, and modulation of the neurotrophic factor system. Multiple studies have reported that SNS treatment is associated with increased levels of MNTs, including 5-hydroxytryptamine (5-HT), dopamine (DA), and norepinephrine (NE) (Chang, 2003; Jin, 2004; Li et al., 2013; Yi et al., 2013; Li and Xu, 2014; Peng, 2014; Wang, 2017; Ni, 2019; Li et al., 2021; Yao et al., 2022; Fang et al., 2023; Yang, 2023; Du et al., 2025). These correlative findings suggest that modulation of monoaminergic systems may contribute to the behavioral effects of SNS. Several studies have observed that SNS treatment correlates with reduced levels of HPA axis-related hormones, including corticosterone, adrenocorticotropic hormone, and corticotropin-releasing hormone (CRH) (Chang, 2003; Jin, 2004; Yu and Jin, 2009; Yi et al., 2013; Peng, 2014; Wei et al., 2016; Wang, 2017; Li et al., 2021; Cai et al., 2025). A number of studies have reported that SNS treatment correlates with changes in oxidative stress markers such as increased superoxide dismutase activity and decreased malondialdehyde levels and mitochondrial function adenosine triphosphate levels (Cui, 2021; Zhang et al., 2023; Du et al., 2025). Several studies have observed that SNS treatment is associated with increased expression of brain-derived neurotrophic factor (BDNF) and synaptic plasticity-related proteins (Chang, 2003; Yi et al., 2013; Peng, 2014; Ni, 2019; Su, 2019; Shen et al., 2020; Cui, 2021; Li et al., 2021; Fang et al., 2023; Yang, 2023; Zhang et al., 2023). A number of studies have reported that SNS treatment is associated with reduced pro-inflammatory mediators, including tumor necrosis factor-α, interleukin-1β, and interleukin-6 and inhibiting the activation of NOD-like receptor thermal protein domain-associated protein 3 (Su, 2019; Wang W et al., 2022; Liu, 2024; Cai et al., 2025; Wang et al., 2025).

**TABLE 3:** Molecular and biochemical correlates of *Si-ni-san* for depression.

Study	Regions	Molecular changes observed
Ni 2019	Brain, serum	1. ↑5-HT, P < 0.01 2. ↑BDNF, P < 0.01
Wang 2017	Brain, serum	1. ↓CRH, ACTH P < 0.001 2. ↑NE, 5-HT, DA, P < 0.05
Su 2019	Brain, serum	1. ↓IL-6, TNF-α, P < 0.01 2. ↑BDNF, TrkB, P < 0.01
Cui 2021	Brain	1. ↑SYN, PSD-95, P < 0.05 2. ↑ATP, Mfn2, ↓Drp1, Fis1, P < 0.05
Li et al., 2014	Brain	↑NE, 5-HT, DA, P < 0.05, P < 0.01
Li et al., 2021	Brain, serum	1. ↓CRH, ACTH, CORT, P < 0.01, P < 0.05 2. ↑BDNF, TrKB, 5-HT1AR, GR mRNA, ↓MR mRNA, P < 0.01, P < 0.05 3. ↑5-HT, P < 0.01, P < 0.05
Wang H et al., 2022	Brain	↓IL-1β, IL-18, NLRP3, ASC, Caspase-1, P < 0.01, P < 0.05
Yu and Jin, 2009	Serum	↓CORT, P < 0.05
Fang et al., 2023	Brain, serum	1. ↑NE, 5-HT, DA, P < 0.05, P < 0.001 2. ↑p-ERK1/2, p-CREB, BDNF, P < 0.01
Yao et al., 2022	Brain, serum	1. ↑5-HT, P < 0.05 2. ↓IL-1β, P < 0.05
Peng 2014	Brain, serum	1. ↓ACTH, CORT, P < 0.05, CRH, P < 0.01 2. ↑5-HT, NE, DA, P < 0.01, P < 0.05 3. ↑BDNF, TrKB, P < 0.05
Jin 2004	Brain, serum	1. ↓CORT, P < 0.05 2. ↑5-HT, DA, P < 0.01 3. ↑5-HT1A, mRNA, P < 0.01
Chang 2003	Brain, serum	1. ↓CORT, P < 0.01 2. ↑5-HT, DA, p < 0.01, P < 0.05 3. ↑BDNF, NMDAR1, P < 0.05, P < 0.01
Cai et al., 2025	Serum	1. ↓CRH, ACTH, CORT, P < 0.05, P < 0.01 2. ↓TNF-α, IL-6, NF-κB, NLRP3, Caspase-1, P < 0.01 3. ↓ERK, JNK, p38 MAPK, P < 0.01
Yang 2023	Brain	1. ↑5-HT, NE, P < 0.05 2. ↑p-PI3K/PI3K, p-Akt/AKT, p-mTOR/Mtor, P < 0.05 3. ↑BDNF, P < 0.05
Wang et al., 2025	Brain	1. ↑AKT1, P < 0.01 2. ↓IL-1B, IL-6, P < 0.05
Du et al., 2025	Brain	1. ↓MDA, Bax/Bacl-2, Caspase-3, ↑ATP, SOD, SIRT3/GAPDH, SOD2/GAPDH, P < 0.01 2. ↑5-HT, NE, p < 0.01
Liu 2024	Serum	↓IL-β, P < 0.05
Li et al., 2013	Brain, serum	↑5-HT, P < 0.05
Yi et al., 2013	Brain, serum	1. ↑5-HT, DA, NE, P < 0.05 2. ↓CORT, P < 0.05 3. ↑BDNF, P < 0.05
Wei et al., 2016	Brain	↓ACTH, CORT, P < 0.05, p < 0.01
Shen et al., 2020	Brain	↑Synaptophysin, GAP-43, PSD-95, P < 0.05, P < 0.01
Zhang et al., 2023	Brain	1. ↑GluR2, PSD-95, SYN, P < 0.05, P < 0.01 2. ↑ATP, Mfn2, Fis1, P < 0.01

5-HT, 5-hydroxytryptamine; DA, dopamine; NE, norepinephrine; TNF-α, Tumor necrosis factor-α; IL-1β, Interleukin-1β; IL-6, Interleukin-6; NLRP3, NOD-like receptor thermal protein domain-associated protein 3; NF-κB, Nuclear Factor Kappa-B; BDNF, Brain-derived neurotrophic factor; CRH, Corticotropin-releasing hormone; ACTH, adrenocorticotropic hormone; CORT, cortisol; MDA, malondialdehyde; SOD, superoxide dismutase; GR, glucocorticoid receptor; MR, mineralocorticoid receptor; TrkB, Tyrosine receptor kinase B; CREB, cAMP, Response Element-Binding protein; p-CREB, Phosphorylated CREB; SYN, synaptophysin; PSD-95, Postsynaptic Density protein 95; GAP-43Growth-Associated Protein 43; NMDAR1, N-Methyl-D-Aspartate Receptor subunit 1; GluR2, Glutamate Receptor 2 subunit; ASC, Apoptosis-associated Speck-like protein containing a CARD; Caspase-1, Cysteine-aspartic acid protease 1; ERK, Extracellular Signal-Regulated Kinase; JNK, c-Jun N-terminal Kinase; p38 MAPK, p38 Mitogen-Activated Protein Kinase; PI3K, Phosphatidylinositol 3-Kinase; AKT, Protein Kinase B; mTOR, mammalian target of rapamycin; ATP, adenosine triphosphate; Mfn2, Mitofusin 2; Drp1, Dynamin-related protein 1; Fis1, Mitochondrial fission 1 protein; SIRT3, Sirtuin 3; Bax, Bcl-2-associated X protein; Bcl-2, B-cell lymphoma 2 protein; Caspase-3, Cysteine-aspartic acid protease 3; 5-HT1AR, 5-Hydroxytryptamine receptor 1A; GAPDH, Glyceraldehyde-3-Phosphate Dehydrogenase.

**FIGURE 6:**
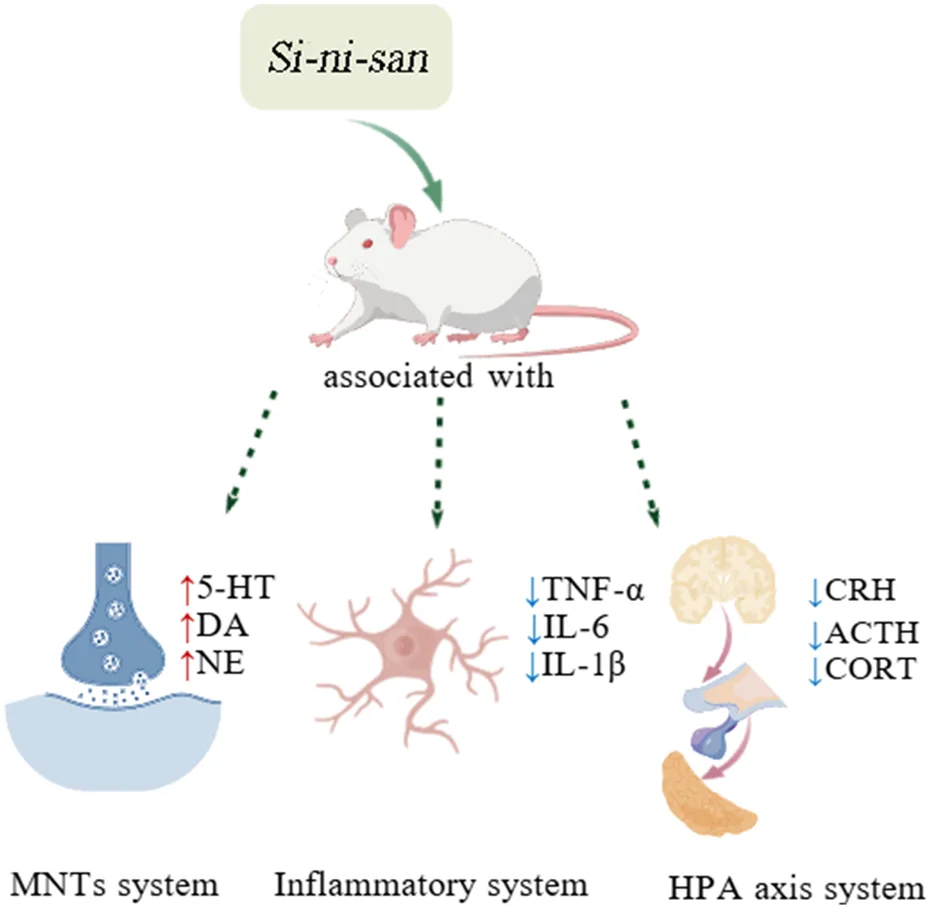
Summary of associated pathways in *Si-ni-san* treatment. Notes: 5-HT, 5-hydroxytryptamine; DA, dopamine; NE, norepinephrine; TNF-α, tumor necrosis factor-α; IL-1β, interleukin-1β; IL-6, interleukin-6; CORT, corticosterone; ACTH, adrenocorticotropic hormone; CRH, corticotropin hormone (Jiang et al., 2025).

### 3.5 Summary of intervention reporting quality

Based on the predefined criteria, the reporting quality of the SNS preparation was generally poor across the 28 included studies. Eight studies (28.5%) were adequately characterized; fifteen (53.5%) were partially characterized; and the remaining five (18%) were inadequately characterized, reporting only the formula name. Formal subgroup analyses to assess whether characterization quality influenced effect estimates were not feasible due to the small number of adequately characterized studies, which precluded meaningful quantitative comparisons. Regarding individual ConPhyMP parameters: batch information was reported in eight studies (29%); pharmacopeial compliance in six studies (21%); and chemical fingerprinting or marker quantification in only five studies (18%). No study employed orthogonal analytical methods for chemical characterization (Supplementary Table S2). Based on the Four Pillars assessment, Pillar 1 (pharmacological requirements) was met by 18 studies (64.2%), Pillar 2 (composition requirements) by eight studies (28.5%), and Pillar 3 (ethical requirements) by 15 studies (53.5%). The specific studies meeting each pillar are detailed in Supplementary Table S3.

### 3.6 Summary of safety outcomes

No included study systematically evaluated drug toxicity, organ toxicity, or drug interactions. Mortality was reported in three studies (10.7%) (Ni, 2019): reported four deaths due to gavage errors and stress intolerance (Shen et al., 2020); stated that all rats survived; and (Cao, 2019) reported one death attributed to malnutrition. None of these deaths were considered drug-related. No study performed histopathological examination of major organs or reported biochemical toxicity markers. Overall, safety data were absent or insufficiently reported across all included studies.

## 4 Discussion

### 4.1 Principal findings

To evaluate the pharmacological effects and underlying mechanisms of SNS in rodent models of depression, this systematic review and meta-analysis incorporated data from 28 studies involving 384 animals. SNS treatment was consistently associated with favorable changes in depressive-like behaviors, including increased sucrose preference in the SPT and reduced immobility time in both the FST and TST. The included studies focused primarily on depression-related mechanisms, mainly assessing MNTs, HPA axis, and inflammatory factors. In conclusion, the primary value of this study lies in providing a comprehensive, systematic, and quantitative synthesis of the available preclinical evidence.

For the SPT, the primary outcome measure extracted was sucrose preference percentage, which is the standard metric for assessing anhedonia in rodents. However, sucrose consumption is also recognized as a valid measure of anhedonia (Berrio et al., 2024). Of the included studies, 20 reported sucrose preference percentage, while one study (Jin, 2004) reported only absolute sucrose consumption. Because sucrose consumption and preference are highly correlated and both reflect the same hedonic construct, we retained this study in the meta-analysis. The use of SMD as the effect size metric accommodates pooling of different units. To assess the impact of this decision, a leave-one-out sensitivity analysis excluding this study was performed, and no single study was outside the 95% CI (Supplementary Figure S3). The inclusion of FST and TST as BD, rather than depression models, reflects their established role in antidepressant screening (Castagné et al., 2011; Trunnell et al., 2024). These tests measure behavioral responses to acute stress rather than recapitulating the chronic pathology of depression.

The magnitude of SNS’s effects varied considerably depending on experimental conditions. Substantial heterogeneity was observed across all three outcomes, as indicated by *I* ^*2*^ values of 87.4% for the SPT, 84.0% for the FST, and 66.3% for the TST. These high *I* ^*2*^ values contributed to downgrading the certainty of evidence to very low and suggest that the pooled estimates should be interpreted with caution. Animal strain significantly moderated the SPT effect, with SD rats showing larger responses than Wistar rats or C57BL/6 mice, possibly reflecting genetic differences in baseline HPA axis tone (Hlavacova et al., 2006). However, the interpretation of this strain-related difference is complicated by several factors. SD rats are an outbred strain with greater genetic heterogeneity than inbred C57BL/6 mice, which may increase behavioral variability and widen confidence intervals (Yilmazer-Hanke, 2008). Housing conditions, diet, and baseline stress levels also vary between facilities and may interact with strain to influence drug response, yet these factors were inconsistently reported (Crabbe et al., 1999). Thus, while strain appears to contribute to heterogeneity, its effect cannot be separated from other experimental variables. Depression model also contributed to heterogeneity in FST and TST, with the largest effects in LPS and MS + CMS models. The pronounced effect in the LPS model may reflect SNS’s anti-inflammatory properties, as LPS induces depressive-like behavior through neuroinflammation (O'Connor et al., 2009), and SNS has been shown to reduce pro-inflammatory cytokines. The large effect in the MS + CMS model, though notable, is based on a single study and should be interpreted with caution. It may reflect the compounded stress burden of early-life adversity combined with chronic stress in adulthood (Shen et al., 2020). However, the wide variation in effect sizes across different models suggests that the magnitude of SNS’s effects is highly dependent on the specific stress paradigm used. Interestingly, lower doses of SNS (<10 g/kg) appeared to produce larger effects than higher doses in the TST, raising the possibility of a non-monotonic dose-response relationship, an observation that warrants careful dose optimization in future translational studies. The high variability in SPT protocols across studies including differences in deprivation period, test duration, and sucrose concentration has been shown to directly affect baseline preference values and subsequent effect size estimates (Primo et al., 2023). Similarly, a study of CMS identified stress duration and severity as major contributors to model variability and treatment response heterogeneity (He et al., 2020). These methodological inconsistencies across studies limit comparability of effect sizes and underscore the need for standardized behavioral testing protocols. Finally, formulation variability such as preparation type, different extraction solvents, and unstandardized ratios remains a critical but often underappreciated contributor, as without chemical fingerprinting, meaningful dose comparisons across studies are difficult. In summary, SNS treatment was associated with antidepressant-like behavioral changes in most included studies, though the magnitude and consistency of these effects varied considerably across experimental conditions. Given the substantial unexplained heterogeneity and the resulting very low certainty of evidence, these findings should be considered exploratory and hypothesis-generating rather than confirmatory. Future studies should adopt standardized protocols to reduce heterogeneity and improve comparability.

Trim-and-fill analyses suggested that publication bias may have led to a modest overestimation of the SPT effect size, whereas for the FST, no missing studies were detected and the adjusted effect size remained unchanged. The GRADE assessment rated the certainty of evidence as very low for all outcomes due to serious inconsistency (substantial unexplained heterogeneity), indirectness of animal-to-human extrapolation, imprecision (small sample sizes), risk of bias, and risk of bias. Therefore, these findings should be viewed as hypothesis-generating, and the precise effect sizes should be interpreted with caution rather than as precise or stable estimates.

A central limitation of this systematic review is the substantial heterogeneity in SNS preparation and phytochemical characterization across the included studies. Only 8 of 28 studies (28.5%) adequately characterized the SNS preparation according to ConPhyMP criteria, and merely 5 studies (18%) reported chemical fingerprinting or marker quantification. Notably, no study employed orthogonal analytical methods. This means that even when the same nominal dosage was used, the actual chemical composition of the administered extract may have varied substantially across studies due to differences in extraction solvents, herbal sources, processing methods, and quality control standards. Without chemical fingerprinting, it is impossible to verify that different studies tested comparable interventions, and the observed differences in effect sizes may reflect variability in what was actually administered rather than true pharmacological effects. Consequently, the pooled effect sizes must be interpreted as average estimates across chemically diverse preparations rather than as stable measures of a defined intervention. This limitation fundamentally affects the validity of our meta-analysis and underscores that the current evidence does not yet permit definitive conclusions about the antidepressant efficacy of SNS. Standardized reporting according to ConPhyMP guidelines is urgently needed in future preclinical studies to improve transparency, reproducibility, and cross-study comparability. Consistent with this, appraisal against the Four Pillars framework revealed that while most studies demonstrated adequate pharmacological design (Pillar 1: 18/28, 64.2%), substantial deficiencies were observed in botanical characterization (Pillar 2: 8/28, 28.5%) and ethical reporting (Pillar 3: 15/28, 53.5%). This persistent divergence between the face validity of the pharmacological models and the substantive inadequacy of botanical characterization and ethical reporting fundamentally limits the reproducibility and mechanistic interpretability of the antidepressant-like effects reported. None of the 28 included studies evaluated drug toxicity, organ toxicity, behavioral adverse effects, or drug interactions. Only three studies reported mortality, all attributed to non-drug-related causes, and no study performed histopathological examination of major organs. This is particularly concerning given that Bupleuri Radix has been associated with hepatotoxicity (Zhong et al., 2024), Glycyrrhizae Radix with pseudohyperaldosteronism and hypertension (Yoshino and Makino, 2026), and Aurantii Fructus Immaturus with potential cardiovascular effects via synephrine (Sun et al., 2023). Although these concerns have not been evaluated in the specific SNS preparations used in the included studies, they highlight the urgent need for future research to incorporate rigorous safety assessments. Without such data, the translational value of these preclinical findings remains limited.

### 4.2 Potential correlates and associated pathways

To explore potential mechanisms underlying the observed behavioral changes, we examined the associations between SNS treatment and several biological pathways. The antidepressant-like behavioral changes observed with SNS were accompanied by changes in multiple biological pathways. SNS treatment was associated with reduced neuroinflammation, regulation of the HPA axis, and increased monoamine neurotransmitters. Within TCM’s *jun-chen-zuo-shi* framework, Bupleuri Radix and Paeoniae Radix Alba provide core antidepressant effects, while Aurantii Fructus Immaturus and Glycyrrhizae Radix et Rhizoma enhance absorption and harmonize the formula (Liang et al., 2025). A systems pharmacology study suggested that SNS may acts through multi-ingredient, multitarget, and multisystem holistic coordination (Shen et al., 2016). Network pharmacology analysis identified 91 active compounds in SNS interacting with 112 depression-related targets, supporting the multi-ingredient and multi-target feature of SNS (Wang H et al., 2022). A recent randomized, double-blind, placebo-controlled trial reported that SNS significantly reduced Hamilton Depression Rating Scale scores in breast cancer patients with mild to moderate depression, suggesting that the whole formulation may have clinical benefits (Hong et al., 2026).

Neuroinflammation represents a key pathophysiological mechanism in depression (Hassamal, 2023). Previous studies have reported that saikosaponins, paeoniflorin, and liquiritin, possess anti-inflammatory properties in experimental models (Liu et al., 2022; Bai et al., 2023; Wang et al., 2024). Dysregulation of the HPA axis is implicated in the pathophysiology of numerous emotional and cognitive disorders, with excessive HPA axis activity being a key feature in depressive disorder (Keller et al., 2017). Similarly, our study found that the antidepressant-like effect of SNS was associated with the modulation of the HPA axis, consistent with previous reports suggesting that certain SNS constituents, including saikosaponins and paeoniflorin, may influence HPA axis function (Li Y et al., 2020; Chen et al., 2024).

MNTs, including DA, NE, and 5-HT, are crucial signaling molecules in the central nervous system, and their dysregulation is implicated in various neurodegenerative and psychiatric disorders (Baranwal and Chandra, 2018). Similarly, our systematic review and meta-analysis found that SNS treatment was associated with enhanced levels of MNTs. Previous studies have reported that saikosaponins and paeoniflorin may increase monoamine levels (Qiu et al., 2013; Guo et al., 2020). Notably, impaired BDNF signaling is associated with depression-like behaviors (Chiba et al., 2012). Specifically, studies have reported associations with increased BDNF expression for saikosaponin (Wang et al., 2021), paeoniflorin (Chen et al., 2019), and liquiritin (Yan et al., 2025).

### 4.3 Strengths and limitation

This meta-analysis provides a synthesis of the available preclinical evidence on the pharmacological effects of SNS in rodent models of depression. Our meta-analysis was conducted through a comprehensive search across databases, duplicate study screening, and data extraction. The mechanistic analysis provides preliminary preclinical evidence for understanding the pathways through which SNS may be associated with antidepressant-like behavioral effects, though further validation in rigorously designed preclinical and translational studies is required before clinical application can be considered. However, several limitations should be acknowledged. First, substantial heterogeneity across species, models, and protocols remained partly unexplained, and incomplete reporting of key methodological items led to a high proportion of “unclear” SYRCLE ratings, limiting the robustness of the analysis. Second, publication bias was detected for the SPT and FST, and trim-and-fill analysis suggested that effect sizes may have been overestimated. Third, the characterization of the SNS preparation was inadequate in the majority of studies: only 28.5% adequately characterized the intervention according to ConPhyMP criteria, and merely 18% reported chemical fingerprinting, preventing verification that different studies tested comparable interventions. Fourth, no included study systematically evaluated toxicity or adverse effects, and all mechanistic interpretations were derived from correlative biochemical measurements without causal validation. Fifth, the predominance of male animals, restriction to English and Chinese publications, and the inherent indirectness of animal-to-human extrapolation further limit the generalizability and translational relevance of the findings.

## 5 Conclusion

This preclinical meta-analysis found that SNS administration was associated with favorable changes in depression-related behavioral measures in rodent models. However, confidence in these findings is very low due to substantial heterogeneity, risk of bias, publication bias, and inadequate characterization of the botanical preparations. Therefore, while the available animal studies suggest antidepressant-like pharmacological effects, these findings should be considered hypothesis-generating rather than confirmatory, and should not be interpreted as evidence supporting clinical use at this stage. And should not be interpreted as evidence supporting clinical use at this stage. Future studies should use standardized protocols, systematic safety assessments, chemically characterized botanical preparations, and causal validation approaches to strengthen the evidence base before clinical translation can be considered.

## Data Availability

The original contributions presented in the study are included in the article/Supplementary Material, further inquiries can be directed to the corresponding author.
