# Supplementary material for: Effects and potential mechanisms of si-ni-san on animal models of depression: a preclinical systematic review and meta-analysis
Source: Front Pharmacol. 2026 Oct 8;17:1818197. doi: 10.3389/fphar.2026.1818197 (PMC13687422; doi:10.3389/fphar.2026.1818197)
Supplement: Supplementary file 1 [file Table1.docx]

Supplementary Material

Content

[1 Supplementary Search strategy	1](#_Toc236240856)

[2 Supplementary Figure	2](#_Toc236240857)

[2.1 Figure S1.	2](#_Toc236240858)

[2.2 Figure S2.	3](#_Toc236240859)

[2.3 Figure S3.	4](#_Toc236240860)

[3 Supplementary Table	6](#_Toc236240861)

[3.1 Table S1.	6](#_Toc236240862)

[3.2 Table S2.	8](#_Toc236240863)

[3.3 Table S3.	12](#_Toc236240864)

# 1 Supplementary Search strategy

PubMed

(((((SNS[Title/Abstract]) OR (Sini San [Title/Abstract])) OR (Sini[Title/Abstract])) OR (Sini Powder[Title/Abstract])) AND (((((("Depression"[Mesh]) OR (Depressive Symptoms)) OR (Depressive Symptom)) OR (Symptom, Depressive)) OR (Emotional Depression)) OR (Depression, Emotional))) AND (((((((((((((((("Models, Animal"[Mesh]) OR (Animal Model[Title/Abstract])) OR (Animal Models[Title/Abstract])) OR (Model, Animal[Title/Abstract])) OR (Experimental Animal Models[Title/Abstract])) OR (Animal Model, Experimental[Title/Abstract])) OR (Animal Models, Experimental[Title/Abstract])) OR (Experimental Animal Model[Title/Abstract])) OR (Model, Experimental Animal[Title/Abstract])) OR (Models, Experimental Animal[Title/Abstract])) OR (Laboratory Animal Models[Title/Abstract])) OR (Animal Model, Laboratory[Title/Abstract])) OR (Animal Models, Laboratory[Title/Abstract])) OR (Laboratory Animal Model[Title/Abstract])) OR (Model, Laboratory Animal[Title/Abstract])) OR (Models, Laboratory Animal[Title/Abstract]))

Web of Science

Depression (Title) and Animal (Title) and SNS (Title) and Sini San (Title) and Sini (Title)

Embase

Sini AND ('depression'/exp OR depression) AND ('animal'/exp OR animal)

CNKI

(TKA%='抑郁' OR TKA%= '郁病' OR TKA%='郁证') AND (TKA%='四逆散' OR (TKA%='柴胡' AND TKA%='芍药' AND TKA%='枳实' AND TKA%='甘草')) AND (TKA%='动物' OR TKA%='啮齿' OR TKA%='鼠' OR TKA%='兔' OR TKA%='犬' OR TKA%='狗' OR TKA%='鸽' OR TKA%='鹌鹑' OR TKA%='鱼')

WangFang

主题:(抑郁 OR 郁病 OR 郁证) and 主题:(四逆散) and 主题:(动物 OR 啮齿 OR 鼠 OR 犬 OR 狗 OR 鸽 OR 鹌鹑 OR 鱼)

VIP 61

R=(抑郁 OR 郁病 OR 郁证) AND R=(四逆散 OR (柴胡 AND 芍药 AND 枳实 AND 甘草)) AND R=(动物 OR 鼠 OR 犬 OR 啮齿)

SinoMed

(((("啮齿"[常用字段:智能]) OR "动物"[常用字段:智能]) OR "狗"[常用字段:智能]) OR "犬"[常用字段:智能]) AND ("四逆散"[常用字段:智能]) AND ((("抑郁"[常用字段:智能]) OR "郁证"[常用字段:智能]) OR "郁病"[常用字段:智能])

# 2 Supplementary Figure

## 2.1 Figure S1.

**Figure S1.** Sensitivity analysis of *Sinisan* for depression. **(A)** The sucrose preference test; **(B)** The forced swimming test; **(C)** The tail suspension test.

## 2.2 Figure S2.

**Figure S2.** Trim-and-fill analysis of *Sinisan* for depression. **(A)** The sucrose preference test; **(B)** The forced swimming test.

## 2.3 Figure S3.

**Figure S3.** Sensitivity analysis of *Sinisan* for depression in the sucrose preference test.

# 3 Supplementary Table

## 3.1 Table S1.

| Table S1. Subgroup analysis of *Sinisan* for depression. | | | | | | |
| --- | --- | --- | --- | --- | --- | --- |
| Parameter | Subgroup |  | No. studies | SMD [95% CI] | *I^2^* | *P* |
| The sucrose preference test | Animal specie | Rat | 18 | 3.31 [2.46, 4.16] | 88.7% | <0.001 |
|  |  | Mice | 3 | 2.60 [1.19, 4.02] | 75.2% | =0.018 |
|  | Animal strain | SD rat | 13 | 3.98 [2.79, 5.16] | 90.5% | <0.001 |
|  |  | Wistar rat | 5 | 1.90 [1.23, 2.57] | 53.4% | =0.073 |
|  |  | C57BL/6 mice | 3 | 2.60 [1.19, 4.02] | 75.2% | =0.018 |
|  | Animal model or screening paradigm | CMS | 15 | 3.25 [2.30, 4.19] | 88.9% | <0.001 |
|  |  | MS | 4 | 3.64 [1.61, 5.67] | 88.5% | <0.001 |
|  |  | MS+CMS | 1 | 3.03 [1.80, 4.26] | 0.0% | <0.001 |
|  |  | MS+CRS | 1 | 1.71 [0.65, 2.77] | 0.0% | <0.001 |
|  | Dose | ≥ 10 g/kg | 13 | 2.58 [1.81, 3.36] | 80.3% | <0.001 |
|  |  | < 10 g/kg | 8 | 4.04 [2.66, 5.42] | 90.5% | <0.001 |
|  | Treatment duration | ≥21 days | 15 | 3.22 [2.30, 4.13] | 88.1% | <0.001 |
|  |  | < 21 days | 6 | 3.16 [1.76, 4.56] | 87.7% | <0.001 |
| The forced swimming test | Animal specie | Rat | 9 | -2.25 [-3.30, -1.20] | 87.5% | <0.001 |
|  |  | Mice | 8 | -2.16 [-3.06, -1.27] | 78.5% | <0.001 |
|  | Animal strain | SD rat | 7 | -2.06 [-3.03, -1.09] | 80.5% | <0.001 |
|  |  | Wistar rat | 2 | -2.67 [-8.41, -3.08] | 96.7% | <0.001 |
|  |  | KM mice | 2 | -1.74 [-3.80, 0.33] | 86.6% | =0.006 |
|  |  | ICR mice | 2 | -2.22 [-4.37, -0.06] | 82.2% | =0.018 |
|  |  | C57BL/6 mice | 4 | -2.44 [-3.94, -0.94] | 81.3% | =0.001 |
|  | Animal model or screening paradigm | CMS | 6 | -1.90 [-3.16, -0.64] | 87.3% | <0.001 |
|  |  | MS | 4 | -1.82 [-2.97, -0.67] | 77.8% | =0.004 |
|  |  | LSP | 2 | -2.22 [-4.37, -0.06] | 82.2% | =0.018 |
|  |  | MS+CMS | 1 | -5.67 [-7.59, -3.74] | 0.0% | <0.001 |
|  |  | MS+CRS | 1 | -0.96 [-1.90, -0.02] | 0.0% | <0.001 |
|  |  | Acute screening paradigms | 3 | -3.17 [-5.84, -0.49] | 89.8% | <0.001 |
|  | Dose | ≥ 10 g/kg | 12 | -2.10 [-2.84, -1.37] | 81.8% | <0.001 |
|  |  | < 10 g/kg | 5 | -2.50 [-4.34, -0.66] | 89.8% | <0.001 |
|  | Treatment duration | ≥ 21 days | 11 | -2.24 [-3.13, -1.35] | 85.5% | <0.001 |
|  |  | < 21 days | 6 | -2.11 [-3.30, -0.92] | 83.8% | <0.001 |
| The tail suspension test | Animal specie | Rat | 1 | -2.02 [-2.86, -1.18] | 0.0% | <0.001 |
|  |  | Mice | 6 | -2.71 [-3.67, -1.75] | 71.2% | =0.004 |
|  | Animal strain | SD rat | 1 | -2.02 [-2.86, -1.18] | 0.0% | <0.001 |
|  |  | KM mice | 2 | -2.66 [-4.14, -1.17] | 65.8% | =0.087 |
|  |  | ICR mice | 1 | -4.21 [-5.90, -2.51] | 0.0% | <0.001 |
|  |  | C57BL/6 mice | 3 | -2.36 [-3.91, -0.81] | 77.5% | =0.012 |
|  | Animal model or screening paradigm | CMS | 3 | -2.55 [-3.37, -1.72] | 31.7% | =0.231 |
|  |  | LPS | 1 | -4.21 [-5.90, -2.51] | 0.0% | <0.001 |
|  |  | CMS+CRS | 1 | -1.04 [-1.98, -0.09] | 0.0% | <0.001 |
|  |  | Acute screening paradigms | 2 | -2.66 [-4.14, -1.17] | 65.8% | =0.087 |
|  | Dose | ≥ 10 g/kg | 5 | -2.11 [-2.81, -1.40] | 55.0% | =0.064 |
|  |  | < 10 g/kg | 2 | -3.84 [-4.96, -2.71] | 0.0% | =0.569 |
|  | Treatment duration | ≥21 days | 4 | -2.19 [-3.15, -1.22] | 66.3% | =0.031 |
|  |  | < 21 days | 3 | -3.11 [-4.51, -1.71] | 69.0% | =0.040 |

## 3.2 Table S2.

| **Table S2.** **Detailed characterization of *Sinisan* preparations in the included studies.** | | | | | | | | | | | |
| --- | --- | --- | --- | --- | --- | --- | --- | --- | --- | --- | --- |
| Study | Botanical name | Plant part | Formula ratio | Source or manufacturer | Batch information | Authentication or voucher specimen | Preparation or extraction method | Pharmacopeial reference or compliance | Chemical fingerprinting or marker quantification | Analytical methods used | Overall characterization |
| Zhou et al. 2015 | NR | NR | R | R | R | NR | R | NR | NR | NR | Partial |
| Su 2019 | NR | NR | NR | NR | NR | NR | R | NR | NR | NR | Inadequate |
| Wang et al. 2004 | NR | NR | NR | R | NR | NR | R | NR | NR | NR | Inadequate |
| Yi et al. 2013 | R | NR | R | R | NR | R | R | NR | NR | NR | Partial |
| Zhang et al. 2023 | R | R | R | R | R | NR | R | NR | R | R | Adequate |
| Cai et al. 2025 | NR | NR | R | R | R | R | R | R | NR | NR | Adequate |
| Du et al. 2025 | NR | NR | R | R | NR | R | R | NR | NR | NR | Partial |
| Liu 2024 | NR | NR | R | R | NR | R | R | NR | NR | NR | Partial |
| Peng 2014 | NR | NR | R | R | NR | R | R | R | NR | NR | Adequate |
| Hu et al. 2016 | NR | NR | R | R | R | NR | R | NR | NR | NR | Partial |
| Wang 2017 | NR | NR | NR | R | NR | NR | R | NR | NR | NR | Inadequate |
| Ni et al. 2019 | NR | NR | R | R | R | NR | R | R | NR | NR | Adequate |
| Shen et al. 2020 | NR | NR | R | R | NR | R | R | NR | R | R | Adequate |
| Chang 2003 | NR | NR | NR | R | NR | NR | R | NR | NR | NR | Inadequate |
| Jin 2004 | NR | NR | NR | R | NR | NR | R | NR | NR | NR | Inadequate |
| Yu et al. 2009 | NR | NR | R | R | NR | NR | R | NR | NR | NR | Partial |
| Li et al. 2013 | NR | NR | R | R | NR | R | R | R | NR | NR | Adequate |
| Li et al. 2014 | NR | NR | R | R | NR | R | R | NR | NR | NR | Partial |
| Li et al. 2021 | NR | NR | R | R | R | NR | R | NR | NR | NR | Partial |
| Yao et al. 2022 | NR | NR | R | R | NR | NR | R | NR | NR | NR | Partial |
| Wang et al. 2022 | NR | NR | R | R | R | NR | R | NR | NR | NR | Partial |
| Fang et al. 2023 | NR | NR | R | NR | NR | R | R | NR | NR | NR | Partial |
| Yang 2023 | NR | NR | R | R | NR | NR | R | NR | NR | NR | Partial |
| Deng et al. 2022 | NR | NR | R | R | NR | NR | R | NR | R | R | Partial |
| Cui 2021 | NR | NR | R | R | NR | NR | R | NR | NR | NR | Partial |
| Cao 2019 | R | NR | R | R | NR | R | R | R | R | R | Adequate |
| Wang et al. 2025 | NR | NR | R | NR | NR | NR | R | NR | NR | NR | Partial |
| Wei et al. 2016 | R | R | R | R | R | R | R | R | R | R | Adequate |

Notes: NR, no reported, R reported.

## 3.3 Table S3.

| **Table S3.** **Assessment of included studies against the Four Pillars of Best Practice framework (Pillars 1-3).** | | | |
| --- | --- | --- | --- |
| Study | Pillar 1 | Pillar 2 | Pillar 3 |
| Zhou et al. 2015 | Partially compliant | Partially compliant | Non-compliant |
| Su 2019 | Compliant | Non-compliant | Compliant |
| Wang et al. 2004 | Partially compliant | Non-compliant | Non-compliant |
| Yi et al. 2013 | Partially compliant | Partially compliant | Compliant |
| Zhang et al. 2023 | Compliant | Compliant | Compliant |
| Cai et al. 2025 | Compliant | Compliant | Compliant |
| Du et al. 2025 | Compliant | Partially compliant | Compliant |
| Liu 2024 | Partially compliant | Partially compliant | Compliant |
| Peng 2014 | Compliant | Compliant | Non-compliant |
| Hu et al. 2016 | Compliant | Partially compliant | Non-compliant |
| Wang 2017 | Partially compliant | Non-compliant | Compliant |
| Ni et al. 2019 | Partially compliant | Compliant | Non-compliant |
| Shen et al. 2020 | Compliant | Compliant | Compliant |
| Chang 2003 | Partially compliant | Non-compliant | Non-compliant |
| Jin 2004 | Compliant | Non-compliant | Non-compliant |
| Yu et al. 2009 | Partially compliant | Partially compliant | Non-compliant |
| Li et al. 2013 | Partially compliant | Compliant | Non-compliant |
| Li et al. 2014 | Compliant | Partially compliant | Partially compliant |
| Li et al. 2021 | Compliant | Partially compliant | Compliant |
| Yao et al. 2022 | Compliant | Partially compliant | Non-compliant |
| Wang et al. 2022 | Compliant | Partially compliant | Compliant |
| Fang et al. 2023 | Compliant | Partially compliant | Non-compliant |
| Yang 2023 | Partially compliant | Partially compliant | Compliant |
| Deng et al. 2022 | Compliant | Partially compliant | Compliant |
| Cui 2021 | Compliant | Partially compliant | Compliant |
| Cao 2019 | Compliant | Compliant | Non-compliant |
| Wang et al. 2025 | Compliant | Partially compliant | Compliant |
| Wei et al. 2016 | Compliant | Compliant | Compliant |
